# Rare sites of metastases in patients with differentiated thyroid carcinoma and added value of SPECT/CT over planar whole body radioactive iodine scan

**DOI:** 10.1186/s41824-022-00155-0

**Published:** 2022-11-28

**Authors:** Nahla Bashank, Hussein Farghaly, Sara Hassanein, Mohamed Abdel-Tawab, Mohamed Wahman, Hemat Mahmoud

**Affiliations:** 1 Department of Oncology and Nuclear Medicine, Assiut University Hospitals, Assiut University, Assiut, Egypt; 2 Department of Diagnostic Radiology, Assiut University Hospitals, Assiut University, Assiut, Egypt; 3 Department of Clinical Oncology, South Valley University, Qena, Egypt

**Keywords:** Rare metastases, Added value, SPECT/CT, Differentiated thyroid cancer, DTC, Radioactive iodine therapy, Whole body iodine scan

## Abstract

**Background:**

Being aware of the unusual or rare location of thyroid metastases helps in early diagnosis and proper patient management. Rare metastases (RM) can be missed resulting in diagnostic pitfalls and delayed treatment. The use of single-photon emission computed tomography/computed tomography (SPECT/CT) imaging in the follow-up of differentiated thyroid cancer (DTC) patients provides precise anatomical localization and characterization of RM that may be missed or misinterpreted in planar whole body iodine-131 (WBI) scan. There is a lack of knowledge about dealing with such patients, the treatment they should receive, and therapy response due to the rarity of such cases. In this work, we reported these rare cases increasing awareness about them and their methods of treatment with response to therapy and evaluated the added value of SPECT/CT imaging in changing patients’ management.

**Materials and methods:**

In this study we reviewed all patients with DTC referred to our unit either for initial radioactive iodine-131 therapy (RAIT) or under follow-up from January 2019 to January 2022. When a suspected lesion was detected in a conventional planar WBI scan whether follow-up scan or post-therapeutic scan, SPECT/CT was acquired immediately in the same session for that region. Additional imaging modalities were performed for confirmation. Response to the given treatment either disease progression (DP) or favorable response which include complete response (CR), partial regression (PR) and stable disease (SD) recorded for each patient.

**Results:**

Two hundred and forty patients with DTC referred to our unit over a three-year period (from January 2019 to January 2022) were reviewed. Forty patients developed lung and bone distant metastases. Twenty-one patients were thought to have metastases at unusual sites. Due to incomplete data (no SPECT/CT pictures or confirmatory imaging), 6/21 patients were eliminated. We studied 15 patients with RM (9 females, 6 males) with a median age of 52 years (range 27–79). All patients received the initial RAIT after thyroidectomy in addition to other therapeutic modalities, e.g., radiotherapy (RTH), chemotherapy (CTH) or surgical tumor excision after detection of RM. Ten out of 15 patients (66.67%) showed favorable response to therapy (2 patients had CR, 6 patients had PR and 2 patients had SD), whereas only 5 patients had DP. Additional SPECT/CT changed management in 10/15 patients (66, 67%) of patients.

**Conclusion:**

RM identification is mandatory to avoid misdiagnosis and delayed therapy. Increasing the awareness about such rare cases allows for better management. SPECT/CT could significantly impact patients' management through its precise anatomic localization and lesion characterization.

## Background

About 7 to 23% of patients with differentiated thyroid carcinoma (DTC) have distant metastases either at the time of diagnosis or develop distant metastases later on during follow-up (Kunadharaju et al. 2015). Lungs and bones are the most common sites for distant metastases in patients with DTC, while the liver, brain, adrenals, kidneys, pancreas, esophagus, endotracheal, muscles and eye are considered rare sites for thyroid metastases (Chattopadhyay et al. 2012; Farina et al. 2016; Palaniswamy and Subramanyam 2018a; Portela et al. 2015; Song et al. 2012). Although bone metastases are common, solitary metastasis to bone in DTC patients is considered rare. The rate of bone metastases is higher for follicular thyroid carcinoma (FTC) (7–28%) than papillary thyroid carcinoma (PTC) (1–7%) which is considered a rare condition also. This is explained by FTC’s tendency to spread hematogenously, while PTC tends to spread through the lymphatic system. Rare metastases (RM) can be missed resulting in diagnostic pitfalls and delayed treatment (Farina et al. 2016). There is a lack of knowledge about dealing with such patients, which treatment they should receive, and the therapy response due to the rarity of such cases. More studies are needed to establish a therapeutic algorithm with accurate and definitive treatment for these patients (Djenic et al. 2015; Yoon et al. 2020; Zunino et al. 2019). Treatment options include total thyroidectomy followed by radioactive iodine-131 therapy (RAIT) with external beam radiotherapy (RTH) and systemic therapy in selected patients (Kunadharaju et al. 2015). Radioactive I-131 used for decades for diagnosis and therapy in patients with DTC (Haugen 2017). Diagnostic or post-therapy whole body iodine-131(WBI) scan with other imaging modalities computed tomography (CT), ultrasonography (US), conventional magnetic resonance imaging (MRI) and Diffusion MRI and laboratory tests (serum thyroglobulin (Tg) and anti-thyroglobulin (antiTg) antibodies levels evaluation) during follow-up of patients with DTC are helpful in early diagnosis of such RM (Rathinam et al. 2017).

Addition of single-photon emission computed tomography/computed tomography (SPECT/CT) to conventional planar WBI scan also increases the incidence of RM detection by precise anatomical localization and characterization of such lesions. SPECT/CT can discriminate suspicious pathological uptake and RM from physiological benign uptake in GIT, urinary tract, salivary glands as well as in the liver (Palaniswamy and Subramanyam 2018b). SPECT/CT provides information about the site, size and avidity of lesions with follow-up of each lesion separately with prediction and assessment of therapy response thus affecting therapy decision-making (Spanu et al. 2021). RM identification could have a significant impact on patients' management, e.g., avoiding inappropriate treatment or unnecessary surgery due to misdiagnosis, facilitating therapy with favorable response if discovered early, or modifying therapy decision-making by adding other modalities of therapy like RTH or CTH (Farina et al. 2016).

Our study aims to study and report these rare cases of distant metastases in patients with DTC to increase the awareness about them and their methods of treatment with response to therapy.

## Patients and methods

In this study we reviewed all patients with DTC referred to our unit (nuclear medicine unit, Assiut University Hospitals) from January 2019 to January 2022 for RAIT after total or near-total thyroidectomy with/without lymph nodes neck dissection or under follow-up. When focal radioactive iodine uptake suspicious for distant metastases detected in conventional planar WBI scan, additional SPECT/CT imaging for that lesion was acquired in the same session. Confirmation by other radiological modalities was considered. Patients without SPECT/CT images or confirmatory investigations were excluded. We considered the detected metastatic lesions as RM if they are in an unusual site for metastases (occurring in less than 1–5% of DTC patients) or previously documented as a case report. Histopathological diagnosis, laboratory, imaging and clinical data were reviewed. Patients were classified into (stages I, II, III and IV) according to the 8th edition; American Joint Committee on Cancer (AJCC) TNM staging system of DTC (Tam et al. 2018) and risk stratified into (low, intermediate and high risk) according to the 2015 American Thyroid Association (ATA) Management Guidelines for adult patients with DTC (Haugen 2017). In addition to RAIT, other therapeutic modalities (external beam RTH, CTH and surgical tumor excision) were applied according to each case. RM found in patients within 6 months of initial diagnosis were classified as (synchronous) and those developed later on during follow-up classified as (metachronous). RM was detected either by diagnostic follow-up WBI scan or post-therapy scan with the addition of SPECT/CT. Diagnosis was confirmed by other radiological modalities (CT, MRI, Doppler and the US) and biopsy with histopathological examination if possible in certain cases. Tumor Markers (Tg and antiTg antibodies) before and after treatment of RM were recorded. We classified disease response to the given treatment either into disease progression (DP) or favorable response which include complete response (CR), partial regression (PR) and stable disease (SD).

Follow-up WBI scan was acquired 48 h after oral intake of 3–5 mCi of I-131 (as I-123 is not available in our country), while post-therapeutic WBI scan was acquired (3–7 days) after therapeutic dose of radioactive iodine. Planar WBI scan was first obtained in the anterior and posterior projections using dual-head γ-camera (Siemens Healthineers, Symbia Intevo SPECT/CT) with parallel-hole high energy collimators, using a 20% energy window set at 364 keV. If any suspected lesion was detected in planar WBI scan, additional SPECT/CT was acquired immediately for that region in the same session. A total of 64 frames were acquired in a step and shoot mode (25 s ⁄ stop) for follow-up WBI scan and (10 s⁄stop) for post-therapeutic WBI scan, in a non-circular 360° arc and a matrix size of 128 × 128. After SPECT acquisition, a low dose CT without contrast was acquired for anatomical mapping and attenuation correction. The CT tube voltage was 130 keV with tube current 30 mA. The total study duration was approximately 30 min.

Statistical analysis was performed using SPSS (version 21: SPSS Inc., Chicago II). Quantitative variables were expressed as means ± SD; qualitative data were expressed as percentage.

## Results

Over a 3-year period (from January 2019 to January 2022), 240 patients with DTC referred to our unit were examined. Distant metastases were found in 40 patients. From these patients, 21 patients were suspected to have RM on planar I-131 WBS. Six patients were excluded from our series due to insufficient data (lack of SPECT/CT images or confirmatory imaging). A total of 15 patients were eligible for our study (9 females, 6 males) with a median age of 52 years (range27-79 mean 55.8 ± 15.14). Out of these fifteen cases, eight patients (53.3%) had PTC (3 of them were follicular variant subtype) and seven patients (46.7%) had FTC, one of them had poorly differentiated FTC (insular subtype).Six patients (40%) initially presented with a neck swelling while nine patients (60%) presented with unusual symptoms related to metastatic lesions at the time of diagnosis (shoulder swelling in 3 patients, abnormalities in vision in 2 patients, headache due to brain lesion in 2 patients, iliac bone mass and dyspnea). According to the American Thyroid Association (ATA) risk-stratification system, 13 of our patients (86.7%) were high risk and only two patients were an intermediate risk. Six patients were less than the age of 55, two of them were considered stage I, and four patients were considered stage II according to the eighth edition of the AJCC/TNM cancer staging system, while the other nine patients (60%) were more than the age of 55 and all were considered stage IV. All patients received the initial therapy (total or near total thyroidectomy with/without lymph node dissection followed by RAIT) in addition to external beam radiotherapy (RTH) for RM treatment in 8 patients (53.33%) and surgical excision in two patients. Most patients received multiple doses of radioactive iodine with a median of 4 doses (range 1:5 doses mean 2.67 ± 1) with cumulative dose ranges between 175 and 850 mCi (mean 426.13 ± 171.17 mCi) over a period of 5 years and a half (range 1.5–7 years mean 3.2 ± 1.8 years).

Response of the patients to therapy was variable with only 1/3 of patients (5/15) showed DP. The worst prognosis was noted in patients with brain metastases and in cases resistant to radioactive iodine therapy and death occurred from disease progression, while parapharyngeal space metastatic cases (2/15) patients (13.3%) showed CR with no evidence of residual disease and complete resolution of RM lesion. The rest of the patients (8/15) showed PR and SD. Twelve (12/15) patients (80%) were alive (ten patients with persistent disease and two showed complete remission) while death occurred in only three patients (20%), from disease progression or the presence of RM in a vital site (brain and endotracheal metastases) as illustrated in Table 1.

**Table 1:** Patients’ characteristics: including staging, risk stratification and treatment details

Cases	Age	Sex	Presentation	Histologic type	TNM staging (AJCC, 2018)		Risk stratification	Distant metastasis	Cumulative dose (mCi)	Follow-up duration (years)	Disease status	Last observation
1	60	F	Shoulder swelling	FTC	T1bN0M1	IVb	High	Bone and lung	850	5	Regression with complete resolution of RM	Alive with disease
2	59	F	Diminution of vision and Dorsal mass with both LL weakness	FTC	T3N0M1	IVb	High	Bone	400	2	Progression	Alive with disease
3	45	F	Neck swelling	FTC	T3NxM1	II	High	Lung Muscle Renal	600	7	Progression	Alive with disease
4	32	F	Neck swelling	PTC (classic variant)	T4aN1M0	I	Intermediate	No	375	2.5	Complete resolution	Alive free from disease
5	55	M	Iliac mass	FTC	Unknown	IVb	High	Bone	400	3	Stable	Died
6	60	F	Shoulder swelling	FTC	T3bN1M1	IVb	High	Bone, lung and brain	600	4	Stable	Alive with disease
7	42	F	Neck swelling	PTC (classic variant)	T2N0M1	II	High	Bone	300	1	Regression	Alive with disease
8	75	M	Eye scotoma with choroid mass in the eye	FTC	Unknown	IVb	High	Bone, lung, liver and eye	470	3	Regression	Alive with disease
9	79	F	Neck swelling	Insular (poorly differentiated)	T4bN0M0	IVa	High	No	180	3	Regression	Alive with disease
10	75	F	Shoulder swelling	PTC (classic variant	T2N1M1	IVb	High	Lung bone	450	3	Regression	Alive with disease
11	64	M	Neck swelling	PTC (unknown)	T4aN1M1	IVb	High	Renal	470	2.5	Progression	Alive with disease
12	53	M	Solitary brain lesion	PTC (follicular variant)	Unknown	II	High	No	150	1	Regression	Alive with disease
13	48	M	Solitary brain lesion (headache)	PTC (follicular variant)	T1bN0M1	II	High	Bone, lung	450	2	Progression	Died
14	27	F	Neck swelling	PTC (follicular variant)	T1bN1M0	I	Intermediate	No	175	1.5	Complete resolution	Alive free from disease
15	63	M	Dyspnea	PTC (classic variant)	Unknown	IVb	High	Bone trachea	375	7	Progression	Died

*FTC* follicular thyroid carcinoma, *PTC* papillary thyroid carcinoma

Sixty percentage of RM cases (9/15) were synchronous, while 40% were metachronous (ranging from 1 to 20 years). In (13/15) patients (86.66%) were symptomatic and symptoms related to RM mass effect, while in (2/15) patients (13.33%) were asymptomatic and RM was discovered accidentally during routine follow-up imaging. RM lesions were found in the following sites: kidney and pancreas, sella turcica, eye, liver, temporalis and pterygoid muscles, parapharyngeal space, solitary brain metastasis, solitary bone metastasis, endotracheal and malignant jugular vein thrombosis. All the lesions were discovered by SPECT/CT after diagnostic or post-therapeutic WBI scans with confirmation by additional radiologic imaging modalities (CT, MRI, US, PET/CT and Colored Doppler) to avoid false positive results. All RM were iodine avid and treated by multiple doses of RAIT. After RM diagnosis, external beam RTH was added in 8 patients (53.33%) for treatment of 4 intracranial, 3 bony, and endotracheal lesions. Brain surgery was not recommended by neurosurgeons in three patients due to poor prognosis and multi-organ metastases. External beam radiotherapy RTH was applied instead of surgery for palliation. Brain surgery was applied in one case as a curative therapy in combination with RAIT and the patient showed favorable response to treatment. Combined chemo-radiotherapy was added in one case that showed resistance to RAIT. Characteristics of RM with laboratory details before and after treatment are described in Table 2.

**Table 2:** Rare metastatic (RM) lesions' characteristics with laboratory details

Cases	RM site	RM appearance time	RM symptoms	TM before RM treatment (ng/ml)		TM after RM treatment (ng/ml)	RM imaging	RM treatment
1	Kidney and pancreas	Synchronous	No	Tg AntiTg	2320 1.87(negative)	285 0.9	Fu WBIs CT MRI	RAIT
2	Sella turcica	Synchronous	Diminution of vision	Tg AntiTg	> 500 0.2	3544 < 15 (negative)	PT WBIs MRI	RAIT + Brain RTH
3	Muscle	Metachronous (4 years After thyroidectomy)	Neck swelling	Tg AntiTg	964 26.5 (negative)	> 500 13.5 (negative)	Fu WBIs PET/CT	RAIT
4	Parapharyngeal mass	Synchronous	Neck swelling	Tg AntiTg	3.26 267 (positive)	0.18 20 (positive)	PT WBIs MRI	RAIT
5	Solitary bone (Iliac mass)	Metachronous (5 years After thyroidectomy)	Iliac mass	Tg AntiTg	> 3000 0.5	> 300 55 (negative)	Fu WBIs CT MRI	RAIT + RTH
6	Solitary brain	Metachronous (1 year After thyroidectomy)	Headache	Tg AntiTg	> 2500 385 (positive)	> 500 337	Fu WBIs MRI	RAIT + Brain RTH
7	Bone metastasis from PTC	Synchronous	Dorsal mass	Tg AntiTg	57 105 (positive)	2.14 207	Fu WBIs CT	RAIT + RTH
8	Eye and liver	Metachronous (20 years after thyroidectomy)	Flashes of light Black spot in visual field	Tg AntiTg	Unknown	Unknown	Fu WBIs Ocular CT PET/CT	RAIT
9	IJV malignant thrombosis	Synchronous	No	Tg AntiTg	1867.5 0.5	7.5 19.5 (negative)	Fu WBIs Colored Doppler	RAIT + excision
10	Bone metastasis from PTC	Synchronous	Shoulder swelling	Tg AntiTg	> 500 15(negative)	11 (negative)	PT WBIs CT	RAIT + RTH
11	Renal	Metachronous 2 years after thyroidectomy)	Neck swelling	Tg AntiTg	965 0.78(negative)	4000 (negative)	Fu WBIs MRI	RAIT
12	Brain	Synchronous	Headache Brain SOL	Tg AntiTg	1278 0.7(negative)	930 (negative)	Fu WBIs MRI	RAIT + Brain RTH excision
13	Brain and liver	Synchronous	Headache HFL and Brain SOL	Tg AntiTg	9400 10 (negative)	13,888 (negative)	Fu WBIs MRI Abdominal US	RAIT + Brain RTH
14	Parapharyngeal	Synchronous	Neck swelling	Tg AntiTg	197 17.8(negative)	0.84 (negative)	PT WBIs	RAIT
15	Endotracheal	Metachronous (4 years after thyroidectomy)	Dyspnea	Tg AntiTg	186 10.37(negative)	7120 (negative)	PT WBIs MRI PET/CT	RAIT + RTH + CTH

*RM* rare metastasis, *TM* tumor marker, *Tg* Thyroglobulin, *AntiTg* anti-thyroglobulin antibodies, *Fu* WBIs follow-up whole body iodine-131 scan, *PT* WBIs post-therapy whole body iodine-131 scan, *RAIT* radioactive iodine-131 therapy, *RTH* radiotherapy

Adding SPECT/CT changed patients’ management in 66.67% (10/15) of our patients by preventing unnecessary horrible surgery in two patients misdiagnosed as eye melanoma and pituitary adenoma by MRI, adding RTH in 8 patients and facilitating surgery (excision of IJV thrombus, brain tumor excision) in two patients. In 33.33% of patients, management not changed as RAIT was described from the start as shown in Table 3.

**Table 3:** Change in patients’ management after addition of SPECT/CT for characterization of RM lesion

Cases	*Misdiagnosis before SPECT/CT	After additional SPECT/CT	Management changed
1	Bone metastases/colonic activity	Kidney and pancreas metastases	No
2	Head lesion (pituitary lesion/skull metastases)	Metastasis in Sella turcica	Prevent unnecessary surgery + RTH
3	Salivary activity	Muscle metastasis	No
4	Cervical LN	Para pharyngeal mass	No
5	Colonic activity/contamination	Solitary iliac bony mass	+ RTH
6	Head lesion (bone /brain)	Solitary brain metastasis	+ RTH
7	Chest lesion (Lung /bone)	Bone metastases from PTC	+ RTH
8	Eye lesion (Eye melanoma/metastases)	Eye metastasis	Prevent surgery (eye enucleation)
9	Cervical LN	IJV malignant thrombosis	+ surgical excision
10	Chest lesion (Lung /bone)	Bone metastasis from PTC	+ RTH
11	Physiological renal activity	Renal metastases	NO
12	Primary brain tumor	Solitary Brain metastasis	+ surgical excision Brain RTH
13	Metastases of unknown origin	DTC with iodine avid Brain and liver metastases	RAIT + Brain RTH
14	Cervical LNs	Para Pharyngeal LN	NO
15	Thyroid residual	Endotracheal metastasis	+ RTH and CTH

^*^Misdiagnosis occurred before additional SPECT/CT even in planar WBI scan or other diagnostic modalities (CT or MRI)

+ Addition

/Versus

## Discussion

Data on the optimal management of RM from DTC, their influence on morbidity and overall survival remains scarce (Zunino et al. 2019). Being aware of the unusual or rare locations of thyroid metastases helps in early diagnosis and proper patients' management (Madani et al. 2015). The use of SPECT/CT imaging in the follow-up of DTC patients provides precise anatomical localization and characterization of RM that may be missed or misinterpreted in planar WBI scan (Palaniswamy and Subramanyam 2018b).

PTC represents 53.3% in our study population. Zunino et al., and Madani et al., reported nearly similar results which were explained by higher incidence of papillary carcinoma in their population (Zunino et al. 2019; Madani et al. 2015). In contrast to See et al. (See et al. 2017) who found that FTC was more common than PTC and most frequently associated with distant metastasis than papillary carcinoma due to hematogenous spread.

The mean age of our patients at the time of diagnosis was 55.8 years and only two patients were younger than 40. Similar findings were reported in the literature (Dhanani et al. 2021; Lee and Soh 2010).

Female gender represents 60% of our series; this may be explained by higher incidence of DTC in females. Previous meta-analysis showed that male population had higher risk to develop distant metastasis and therefore a poor prognosis compared to females (Vuong et al. 2018).

Most of RM lesions from DTC were found to develop metachronously in previous studies (Yoon et al. 2020; Zunino et al. 2019; Madani et al. 2015). Conversely, in our series 60% of the detected RM lesions were synchronous. This may be explained by recent improvement in lesion detection after use of hybrid imaging. Similar results were observed by Albano who reported 62% synchronous metastases in their study population (Albano et al. 2018, 2019).

In our study, all RM lesions were iodine avid and continued concentrating I-131 throughout the follow-up period and so treated with multiple doses of radioactive iodine as a first choice. In contrast to these results, Albano et al. reported loss of RAI avidity during follow-up, more frequently (50%) of metachronous metastases and 15% of synchronous metastases (Albano et al. 2019). Most of our study population (66.67%) showed favorable response to RAIT, even in cases with multi-organ distant metastases. This indicates that RM does not always represent a poor prognostic factor for disease outcome. Patients who showed bad prognosis with disease progression had lesions in vital organs, e.g., brain, or were resistant to RAIT. These patients were candidate for tyrosine kinase inhibitor therapy which is not available in our region.

From our study, it was noted that the mean of cumulative radio-iodine activities administered in patients with metachronous metastatic lesions was significantly higher than synchronous lesions. Similar results were reported by a previous study by Albano et al., they also observed that total RAI activities administrated, and the total number of doses was significantly higher in metachronous than synchronous metastases (Albano et al. 2019). In addition to RAIT, other systemic and local therapies may be considered including, metastatectomy, external beam RTH and radiofrequency ablation (Djenic et al. 2015; Paspala et al. 2019). This was considered in our patients where surgery was applied to solitary isolated RM in two cases in addition to RAIT and both cases showed disease regression. Complementary treatment with palliative surgery or external beam RTH was added according to each case especially for patients with multi-organ metastases. More future studies recommended by Djenic et al., to establish therapeutic algorithm with accurate and definitive care of such cases with RM (Djenic et al. 2015).

Adding SPECT/CT with RM identification has a significant impact on patients' management. In the present study management changed in 66.67% of patients by adding local therapy (RTH and metastatectomy) or avoiding inappropriate treatment/ horrible surgery such as eye enucleation in case of eye metastasis misdiagnosed as eye melanoma or monitoring therapy without change in management with favorable response to RAIT if discovered early like in pancreatic and kidney metastases.

Although death from thyroid carcinoma is rare, it occurs mainly in metastatic patients with 5-year survival rate of about 15.3% in multi-organ metastasis (Wang et al. 2014). Three patients (20%) in our study died due to disease progression. Two of them had multi-organ metastases in addition to the rare metastatic site. Similar results were published by Yoon et al., who reported 31.6% death rate in their study due to progressive disease (Yoon et al. 2020).

**Fig. 1:**
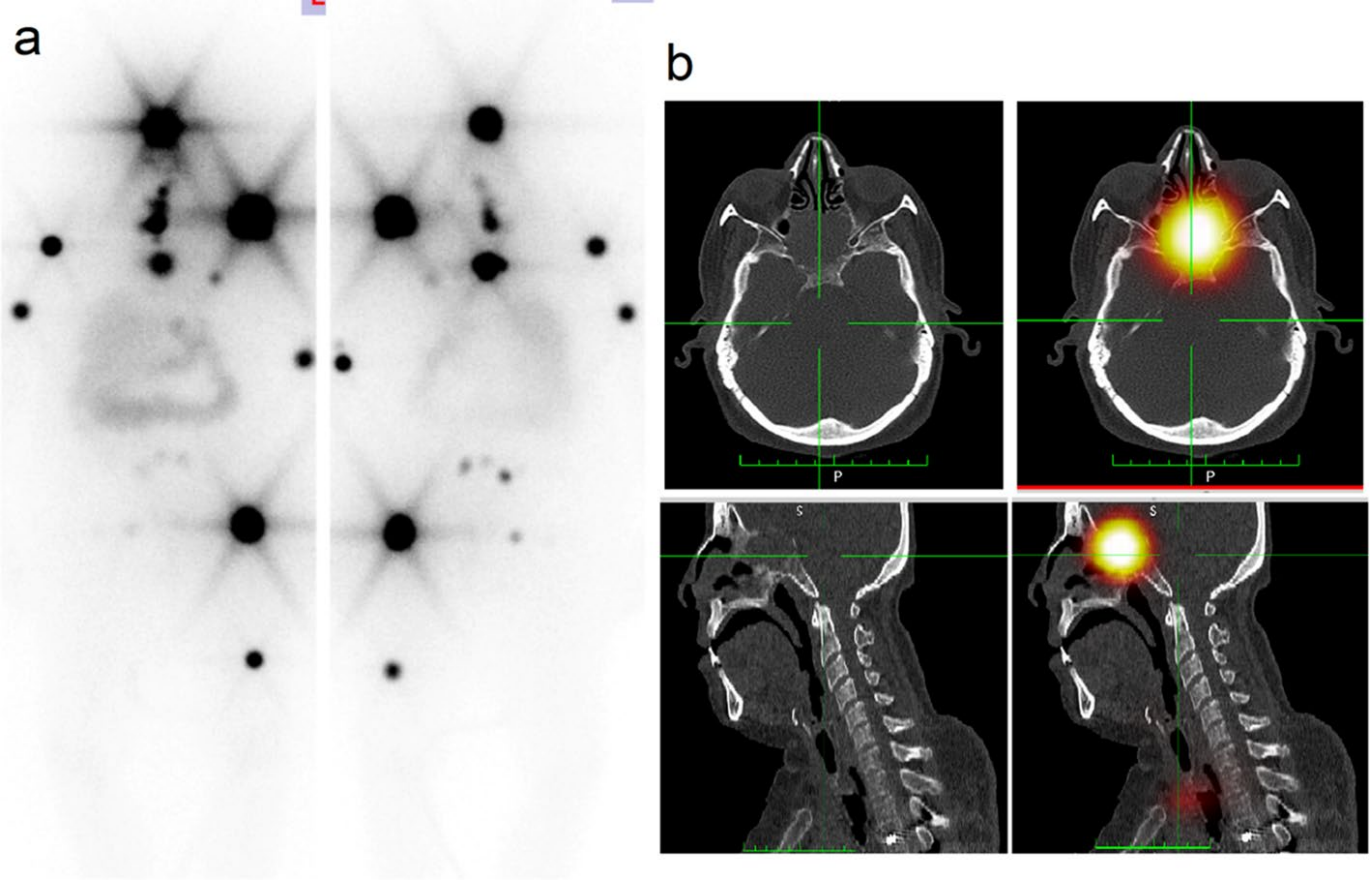
**a** WBI scan showed residual functioning thyroid tissue with multiple iodine avid skeletal metastases and **b** SPECT/CT images showed iodine avid destructive lytic metastatic lesions at sella turcica, 5th and 6th dorsal vertebrae and left humeral head, right humeral shaft and proximal end and mid shaft of left femur

Regarding sites of metastatic disease, metastatic invasion of the skull bone is rare and develops in only 2.5%-5.8% of the cases and mostly affects the sella turcica, pituitary gland, cavernous sinus and sphenoid sinus (Osorio et al. 2017; Sheikh et al. 2018). Herrin we presented a case of sella turcica metastasis, she was treated by RAIT and RTH and still has evidence of residual disease like in our case Fig. 1. The reported incidence of renal metastases is about 3% for the papillary subtype and 6–20% for the follicular subtype (Falzarano et al. 2013; Patel et al. 2011). We reported 2 cases of renal metastases, one of them had papillary and the other had follicular carcinoma.

**Fig. 2:**
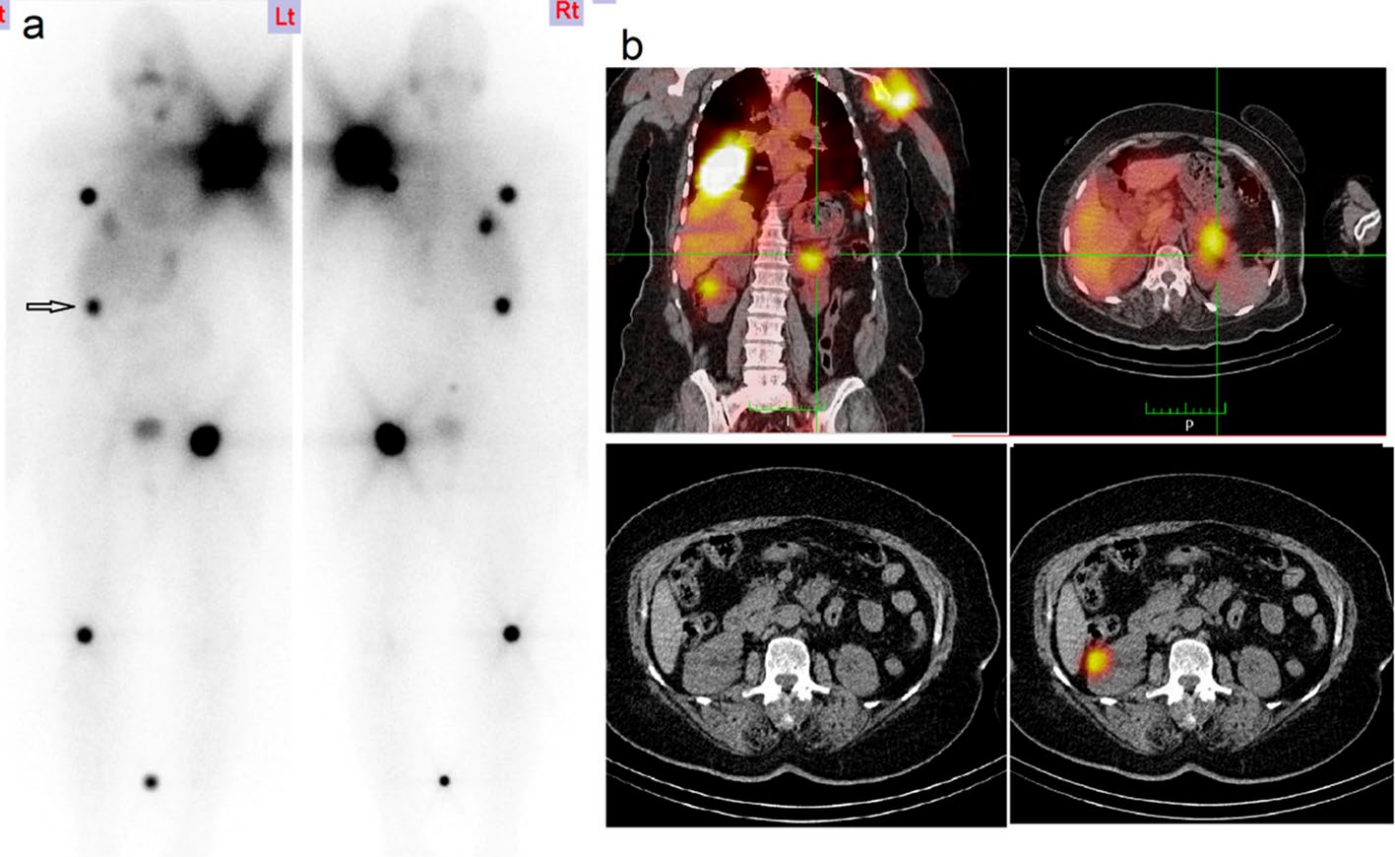
**a** WBI scan showed residual functioning thyroid tissue with multiple iodine avid distant metastatic deposits in the bone, lung and right kidney (arrowed) misinterpreted as bone lesion and missed pancreatic metastases and **b** SPECT/CT images showed iodine avid metastatic deposits in tail of the pancreas (upper images) and the right kidney with slight bulging in the renal cortex (lower images)

Few reported cases of pancreatic metastases from thyroid carcinoma. Most of them derived from papillary thyroid carcinoma. Distant metastases to other organs were encountered in half of these cases (Murakami et al. 2018). Herein we have a case of female patient, FTC who also had bone and lung metastases in addition to renal and pancreatic metastases. She had good response to RAIT Fig. 2.

**Fig. 3:**
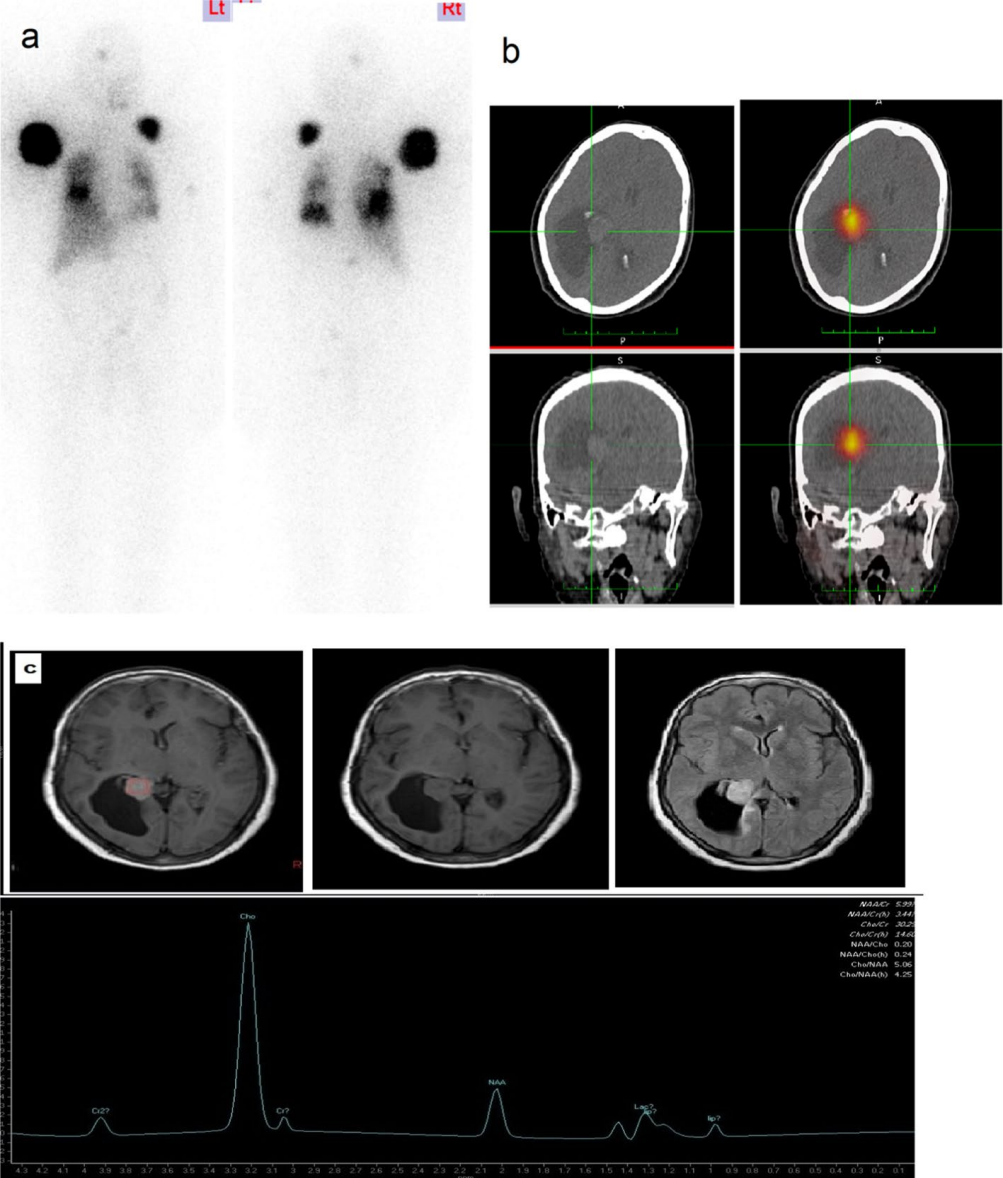
**a** Post-therapy WBI scan shows multiple iodine avid lesions in skull, both shoulders and lung, **b** SPECT/CT image shows iodine avid solitary brain metastasis. **c** MRI conventional brain examination revealed abnormal right intraventricular mass lesion seen at the atrium of the right lateral ventricle being isointense in T1WI, hyperintense in T2WI, FLAIR with homogenous enhancement in post-gadolinium (post-Gd) T1WI, associated focal dilatation of the temporal and occipital horns of the right lateral ventricle with subsequent trans ependymal CSF periventricular edema. MR spectroscopy showed high choline peak, low NAA peak with increased Cho/NAA and Cho/Cr ratios

For DTC, the brain is an unusual site for distant metastases, occurring in about 1% of cases (Kim et al. 2009). By their nature they represent an immediate threat to patients. The uptake of RAI by brain metastatic lesions is low (0–25% of cases) (Lee et al. 2015). In our study we have one case from three cases with solitary brain metastatic lesion that was treated by surgical excision. In accordance with previous studies (Choi et al. 2016; Henriques de Figueiredo et al. 2014), we observed that DTC patients usually presented with other extracranial metastases at time of diagnosis in addition to brain metastases Fig. 3.

Regarding bone metastases from DTC, the rate is higher for FTC (7–28%) than PTC (1–7%). This explained by the tendency of FTC to spread hematogenously. A single bone metastasis in DTC patients is very rare. Palaniswamy et al. reported 3 cases of solitary bone metastases to sacrum, humerus and scapula (12). We reported a case of solitary bone metastasis to iliac bone.

**Fig. 4:**
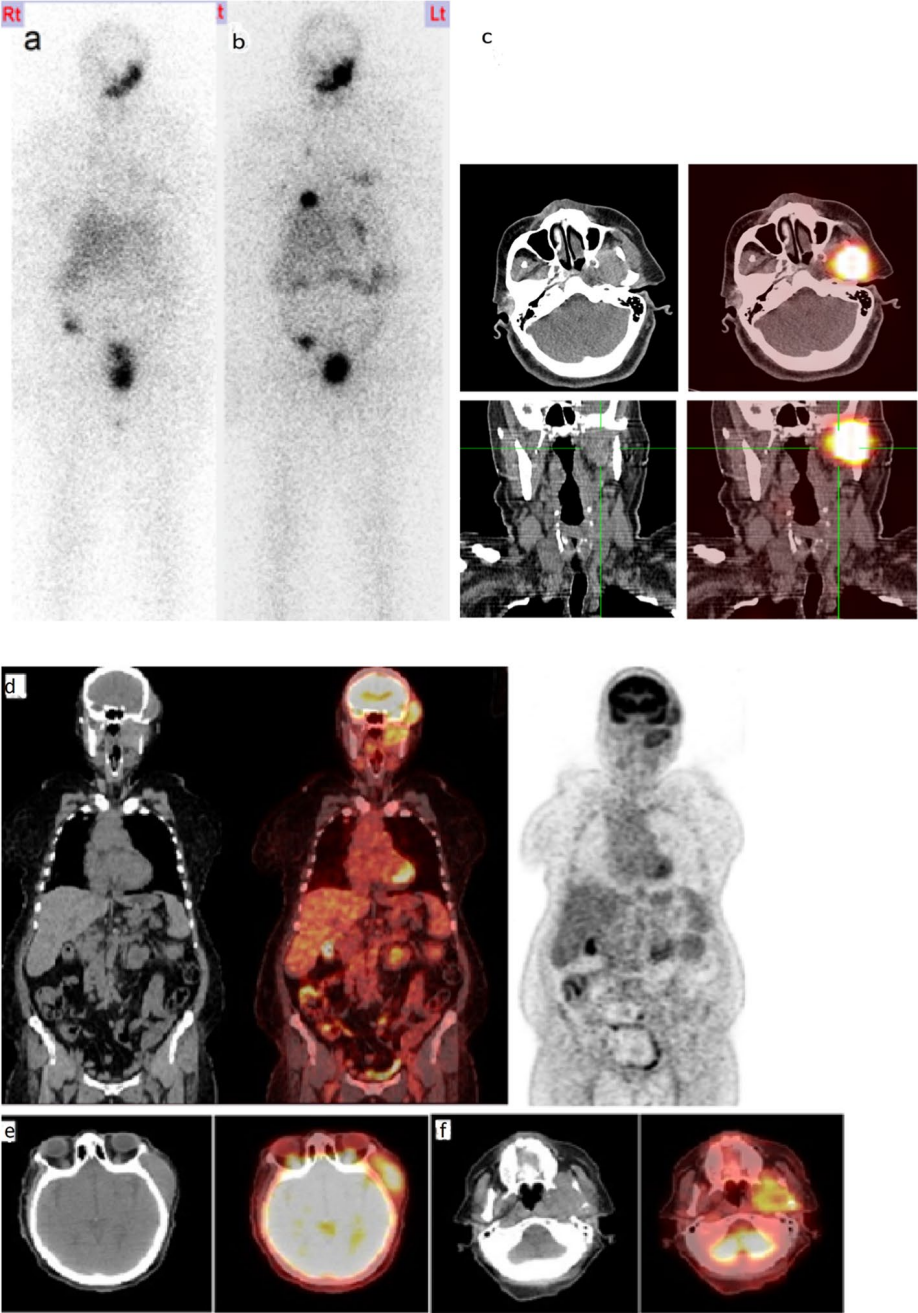
**a** Post-therapy WBI scan showed uptake in left submandibular area misinterpreted as salivary gland activity, **b** follow-up post-therapy WBI scan revealed disease progression with increased uptake in submandibular region and increase in number and intensity of pulmonary lesions, **c** SPECT/CT images show iodine avid pterygoid muscles metastasis. **d** Whole body^18^F FDG-PET/CT coronal images show intense FDG uptake by left temporalis and pterygoid muscles indicating muscle metastases, **e** trans-axial images show increased FDG uptake by left temporalis muscle and **f** trans-axial images show increased FDG uptake by left pterygoid muscles

Only 58 cases of muscular metastasis from DTC have been reported, from 1907 to 2017. The most frequent muscles involved are the gluteus muscles. The majority of muscular metastases are correlated with worse survival (Tunio et al. 2013). In our series we have only one case of skeletal muscle metastases to temporalis and pterygoid muscles, the patient is female with FTC and she also had lung metastases, she received RAIT with progressive disease till now.^18^F FDG-PET /CT was done for confirmation Fig. 4.

Based on the literature, only 112 cases of metastases from DTC to parapharyngeal spaces reported in the last two decades (Giordano et al. 2015). Perfect surgical excision favors response to RAIT with even complete resolution as in our cases.

The existence of eye metastases secondary to primary thyroid neoplasm is an unusual event, with only 22 reported cases from 1979 to 2012, most of them occurred late in the disease course (Avram et al. 2004). In our case choroidal metastasis had occurred 20 years after diagnosis.

### Some limitations need to be addressed

One limitation is small sample size due to the rarity of such cases. Exclusion of rare cases with insufficient data (absence of SPECT/CT images or confirmatory imaging) underestimates the exact number of cases with RM. Unavailability of tyrosine kinase inhibitor drugs in our region rendering assessment of its response on patients with progressive course.

### Recommendation

Furthermore, studies are needed to establish therapeutic algorism in such rare cases and to assess the response to tyrosine kinase inhibitor drugs especially in resistant cases to RAIT.

## Conclusion

RM identification is mandatory to avoid misdiagnosis and delayed therapy, thus facilitating the appropriate therapy with good prognosis if started early. This study increases the awareness about RM lesions from DTC allowing for better management for such cases.

SPECT/CT increases the detection of RM lesions by precise anatomic localization and characterization of lesions. SPECT/CT can change patient management by preventing unnecessary surgery or adding different therapeutic modalities to conventional RAIT, e.g., RTH or CTH.

## Data Availability

All data used in this study can be made available on request.

## Abbreviations

RM: Rare metastasis
DTC: Differentiated thyroid carcinoma
PTC: Papillary thyroid carcinoma
FTC: Follicular thyroid carcinoma
SPECT/CT: Single-photon emission computed tomography/computed tomography
PET/CT: Positron emission tomography/computed tomography
RAIT: Radioactive iodine-131 therapy
RTH: Radiotherapy
CTH: Chemotherapy
EBRTH: External beam radiotherapy
WBI: Whole body iodine-131
CT: Computed tomography
US: Ultrasonography
MRI: Conventional magnetic resonance imaging
Tg: Thyroglobulin
antiTg: Anti-thyroglobulin antibodies
